# A sample cell for the study of enzyme-induced carbonate precipitation at the grain-scale and its implications for biocementation

**DOI:** 10.1038/s41598-021-92235-7

**Published:** 2021-07-01

**Authors:** Jennifer Zehner, Anja Røyne, Pawel Sikorski

**Affiliations:** 1 Department of Physics, Norwegian University of Science and Technology, Trondheim, Norway; 2 The Njord Centre, Department of Physics, University of Oslo, Oslo, Norway

**Keywords:** Biophysics, Climate sciences, Materials science, Biological physics

## Abstract

Biocementation is commonly based on microbial-induced carbonate precipitation (MICP) or enzyme-induced carbonate precipitation (EICP), where biomineralization of $\text {CaCO}_{3}$ in a granular medium is used to produce a sustainable, consolidated porous material. The successful implementation of biocementation in large-scale applications requires detailed knowledge about the micro-scale processes of $\text {CaCO}_{3}$ precipitation and grain consolidation. For this purpose, we present a microscopy sample cell that enables real time and in situ observations of the precipitation of $\text {CaCO}_{3}$ in the presence of sand grains and calcite seeds. In this study, the sample cell is used in combination with confocal laser scanning microscopy (CLSM) which allows the monitoring in situ of local pH during the reaction. The sample cell can be disassembled at the end of the experiment, so that the precipitated crystals can be characterized with Raman microspectroscopy and scanning electron microscopy (SEM) without disturbing the sample. The combination of the real time and in situ monitoring of the precipitation process with the possibility to characterize the precipitated crystals without further sample processing, offers a powerful tool for knowledge-based improvements of biocementation.

## Introduction

Concrete is humanity’s second most consumed material, after water^1^. Cement is used as a binding material in concrete and due to the high demand for concrete and the large emissions of $\text {CO}_{2}$ during the production process of cement, the cement industry accounts for up to 5$\%$ of anthropogenic $\text {CO}_{2}$ emissions^2^. In order to support the global efforts of reducing greenhouse gas emissions, alternative materials need to be developed to cover the demand for construction materials and simultaneously lower the $\text {CO}_{2}$ emissions of the construction industry. One approach to produce a consolidated porous construction material is biocementation, where biomineralization of $\text {CaCO}_{3}$ is utilized for cementation of a granular medium^3^. Comparing concrete materials with same strength, it is suggested that MICP and EICP has the potential to reduce $\text {CO}_{2}$ emissions during the production process^4^.

Calcium carbonates are one of the most abundant minerals on earth, and are the major components of both chalk and limestone. In biomineralization, the most commonly used pathway for inducing $\text {CaCO}_{3}$ precipitation is through the hydrolysis reaction of urea. This hydrolysis reaction can be catalyzed by the enzyme urease^5–7^:

1 $$\begin{aligned} \text {CO(NH}_{2})_{2} + \text {3H}_{2}\text {O} \rightarrow 2 \text {NH}_{4}^{+} + \text {HCO}_{3}^{-} +\text {OH}^{-} \end{aligned}$$

This increases the pH of the solution, which, together with the produced bicarbonate ions, will induce precipitation of $\text {CaCO}_{3}$ if a sufficient amount of calcium is available in the crystallization solution:

2 $$\begin{aligned} \text {Ca}^{2+} + \text {HCO}_{3}^{-} + \text {OH}^{-} \rightarrow \text {CaCO}_{3} \downarrow + \text {H}_{2}\text {O} \end{aligned}$$

The enzyme urease can be produced by microorganisms or extracted from plants^8^. This process described by Reactions 1 and 2 is referred to as microbial-induced carbonate precipitation (MICP) if the enzyme is provided by urease-producing bacteria strains^3,7,9^ or as enzyme induced carbonate precipitation (EICP) if the free urease, for example plant-derived urease, is used to catalyze the hydrolysis of urea^10^. An advantage of EICP is that bacteria cells do not need to be cultivated before or during the reaction, resulting in a simpler production protocol for EICP biocementation. The urea hydrolysis increases the pH, while the precipitation of $\text {CaCO}_{3}$ results in pH decrease. Therefore, the pH is an important parameter for monitoring the biocementation reaction.

Crystallization of a crystal is a two step process, where the nucleation of a crystal is followed by crystal growth. In more general precipitation processes, the amorphous solid might form first and typically transforms to the crystalline phase through a dissolution re-precipitation process. The driving force for nucleation and growth is the difference in chemical potentials of the liquid and the solid crystal. When the chemical potential of the solutes is higher in solution than in the crystal, the solution is said to be supersaturated with respect to the solid phase. $\text {CaCO}_{3}$ has three crystalline polymorphs: calcite, vaterite and aragonite. Additionally, amorphous calcium carbonate can precipitate, which fast transforms in vaterite and calcite. A solution is supersaturated with respect to solid calcium carbonate once $S>1$^11^:

3 $$\begin{aligned} S= \sqrt{\frac{(a_{\text{Ca}^{2+}})(a_{\text{CO}_3^{2-}})}{K_{sp}}} \end{aligned}$$

where $a_{\text{Ca}^{2+}}\cdot a_{\text{CO}_3^{2-}}$ is the ion activity product of calcium and carbonate ions in the solution, and K$_{sp}$ is the solubility product of the nucleating polymorph of $\text {CaCO}_{3}$.

A supersaturated solution is not in a chemical equilibrium, which gives rise to concentration fluctuations in the solution and cluster formation. If a cluster reaches a critical size, a stable nucleus is formed. Typically, the energy barrier for nucleation is significantly lower for nuclei that form on foreign surfaces in the crystallization system than for nuclei that form in the bulk liquid^12^. The former process is referred to as heterogeneous nucleation, and the reduction of the energy barrier depends on the surface tensions between the crystal phase, the liquid phase and the foreign solid substance^12^.

In the production of a biocemented material, several injections of the crystallization solution are often needed in order to achieve sufficient consolidation of the granular material^13–16^. For the first injection in EICP, mainly the sand needs to be considered as nucleation surfaces for heterogeneous nucleation. For MICP, the surfaces of the bacteria may also serve as nucleation sites^5,6^. The initial injections of crystallization solution will lead to the nucleation of calcite crystals within the granular medium. Subsequent injections will then result in growth of the already formed calcite, along with, if the supersaturation becomes sufficiently high, nucleation of additional crystals on the sand surfaces.

The influence of calcite seeds on the pH evolution in small volumes for MICP has been reported previously^17^. Here, it was shown that the presence of calcite seeds in the crystallization system has a significant influence on the average pH of the crystallization solution. Furthermore, the study showed that the calcite precipitation in MICP starts at a lower supersaturation level for seeded samples.

The successful use of MICP for construction depends on a number of interconnected biogeochemical processes. This complexity makes numerical simulations useful tools both for the understanding of the process as a whole and for the design and interpretation of experiments. Simulations are performed over a wide range of scales. Continuum scale models are relevant for field applications, but rely on the use of effective parameters that need to be determined from pore-network and pore-scale models. Direct simulations of processes on the pore-scale allow the use of realistic parameters, but are computationally costly. They also need to be benchmarked against high-resolution pore-scale experiments^18^. Pore-scale experiments for benchmarking should provide information about micro-scale processes, such as precipitation kinetics and spatial and temporal pH distribution during the biocementation process.

MICP/EICP has previously been investigated at the micro-scale using microfluidic chips as model systems for the granular medium^19–21^. Microfluidic chips are commonly made using soft lithography, which is an easily accessible fabrication technique for producing patterned elastomeric polymers, such as PDMS. Using this technique, previous studies have investigated the formation of calcite crystals in MICP at the micro-scale^19^, as well as the effect of bacterial growth and bacteria density on MICP^21^. Furthermore, researchers investigated how multiple injections of the crystallization solution in the microfluidic chip affected the crystallization process, and compared the experimental data with numerical simulations^20^. However, the use of these microfluidic chips does not allow the study of $\text {CaCO}_{3}$ precipitation in the presence of sand surfaces, which is important for benchmarking pore-scale models and consequently for fully understanding the material formation process.

For this purpose, we have developed a microscope sample cell with sand grains and calcite seeds firmly attached. Including crystallization surfaces such as sand and calcite crystals in a microscope setup for real time studies can be challenging, as the particles need to be properly fixed in order not to move during the injections of liquid. The presented sample cell allows us to monitor precipitation of $\text {CaCO}_{3}$ and local changes in pH in the presence of calcite seeds and sand grains, and this can be used for benchmarking pore-sale models. The pH value is a good indicator of the chemical processes that take place during the EICP or MICP. At the early stage of the reaction, when the pH is below 7, the conditions are dominated by the urea hydrolysis. Stable pH in the range 7.0–7.5 at the later stage of the reaction co-insides with the hydrolysis products being consumed by the crystallisation process and indicates that both processes happen at the same rate^17^. To detect local pH differences during the reaction, we use confocal laser scanning microscopy and a previously developed method^17^. Together with ongoing modelling work that involves geochemical approaches, changes in the pH combined with enzyme activity should be sufficient to fully describe the chemical processes in the experimental system under investigation. After completing the microscopy experiments, the precipitated crystals are characterized with Raman microspectroscopy and scanning electron microscopy (SEM). We also investigate how the number of injections of crystallization solution into the sample cell influences the $\text {CaCO}_{3}$ precipitation in the presence of sand.

## Materials and methods

All chemicals were purchased at Sigma-Aldrich (Norway) unless otherwise stated. All solutions were prepared with de-ionized Milli-Q water (DI) and filtered with ${0.22}\, \upmu \text {m}$ polycarbonate syringe-filter before use.

### Urease dilution

Plant-derived urease from *Canavalia ensiformis* (Jack bean) was used to catalyze the urea hydrolysis reaction. The used urease was Type IX powder, with an activity of $50{,}000\, \text {units}\, \text {g}^{-1}$ to $100{,}000\, \text {units}\, \text {g}^{-1}$. Urease powder was dissolved in DI water and added to the crystallization solution. Crystallization solutions with two different urease concentrations ($D_1 = {13}\, \upmu \text {g} \, \text{L}^{-1}$ and $D_2$= ${6}\, \upmu \text {g} \, \text{L}^{-1}$) were used and the samples are in the following denoted as seeds$_{\text {D1}}$ and seeds$_{\text {D2}}$ for experiments with only seeds present and as sand$_{\text {D1}}$ and sand$_{\text {D2}}$ for samples with sand present in the sample cell. The urease stock solution was produced after a standard protocol to ensure comparable enzyme activity and reproducibility of the experiments.

### Crystallization solution

Dissolved chalk solution (DCS) was produced as described previously^22^, and used as a cost-effective calcium source for EICP^13^. Usually, $\text {CaCl}_{2}$ is used in EICP/MICP, however, chloride-ions react fast to form chloride salts and pollute water and can lead to steel corrosion of steel-reinforcement in concrete. Crushed industrial quality limestone (Franzefoss Miljøkalk AS (Norway)) that contained maximum of 0.3 weight percent Mg, was dissolved with 300  mM lactic acid solution. DCS was prepared by dissolving 5 g of crushed limestone in 50 ml of 300  mM lactic acid. After 24 h, the remaining parts of limestone were filtered out of the solution with a $0.22\, \upmu \text {m}$ syringe-filter. DCS, produced with the above described procedure, had a calcium concentration of 122 mM. The calcium concentration of DCS was measured with potentiometric titration with EDTA. Titration was carried out automatically via Mettler Toledo G20 titration setup with a dynamic titration mode.

Urea was added to the dissolved chalk solution, so that the starting concentration of urea in the solution was 0.1 M. The staring pH value of the crystallization solution was 5.3 pH units. To start the reaction, plant derived urease solution was added to the solution, mixed thoroughly, and injected into the sample cell. In the following this mixture of DCS, urea and urease is referred to as crystallization solution. The crystallization solution was added to the sample cell approximately 10 s after adding the urease enzyme. No precipitation of $\text {CaCO}_{3}$ is expected at such early stages of the hydrolysis reaction due to the low starting pH value of the crystallization solution. In the presented experiments single and multiple injections of the crystallization solution in the sample cell were investigated. For the multiple injection experiment the reaction was stopped after 45 min with ethanol after each injection, as at this time point all crystal nucleation was completed. All liquid was removed from the sample cell and the sample cell was flushed with DI water and dried before the next injection of crystallization solution.

### Sample cell preparation

For the sample cell, Ibidi polymer slides and Ibidi sticky-Slide VI 0.4 were used (Ibidi GmbH, Gräfelfing, Germany). The Ibidi sticky-Slide VI 0.4 is a bottomless six channel slide with a self-adhesive underside where the polymer slide can be attached. This results in a slide with six sample cells. The sample volume for one cell is ${30}\, \upmu \text {L}$ and each channel is 17 mm long, 3.8 mm wide, and 0.4 mm high.

**Figure 1:**
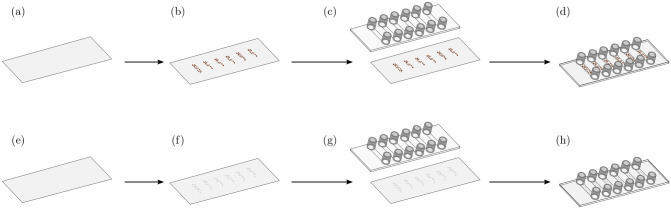
Fabrication and assembly process of sample cells with incorporated calcite seeds (**a**–**d**) and sand grains (**e**–**h**). (**a**,**e**) show the Ibidi polymer slide, and the self-adhesive Ibidi sticky-Slide VI 0.4 was added to the polymer slide in (**c**,**g**) for the assembly of the sample cell.

The sample cell was used to investigate two different crystallization conditions: EICP in presence of the calcite seeds, and EICP in the presence of sand grains. Calcite seeds and sand were attached to the polymer slide to avoid movement of seeds and sand during the measurements (Fig. 1). For the first experiment, calcite seeds were directly grown on the surface of the polymer slides. For that, the polymer slide was placed in a beaker with 5 mM $\text {CaCl}_{2}$ and the crystallization reaction was initiated by adding 5 mM $\text {Na}_{2}\text {CO}_{3}$ solution. Areas of the slide that would be outside the channels in the finished sample cell were covered during the crystallization reaction to avoid calcite seeds outside of the channels. The reaction was left to proceed for 3 d. The slides were subsequently washed with DI water and ethanol and dried. The dry polymer slide (Fig.  1b) was attached to a sticky-Slide VI 0.4 (Fig.  1c). The schematic of the finished sample cell with calcite seeds is shown in Fig.  1d, where the calcite seeds are located inside the channels of the sample cell.

For the second experiments, sand grains (50–70 mesh particle size; $210\, \upmu \text {m}$ to ${297}\, \upmu \text {m}$) were attached to the polymer slide (Fig.  1b). The Quartz Sand ($\text {SIO}_{2}$) was purchased at SigmaAldrich (product number: 274739). Sand was placed on the polymer slide, heated up to 200 °C and left for 10 min to thermally attach the sand to the polymer slide. Also sand was attached only to the areas of the polymer slide that would form the bottom of the channels in the sample cell. The sample cell was assembled by attaching the polymer slide to a sticky-Slide VI 0.4 (Fig.  1d,h).

### Local pH monitoring

Local pH changes during the precipitation processes were monitored in real time and in situ on a single crystal level with confocal laser scanning microscopy (CLSM, TCS Leica SP8, objective 40×, NA 1.1) using a method described previously^22^. The pH sensitive fluorescent dye N-(rhodamine 6G)-lactam-ethylenediamine (R6G-EDA, concentration: $180\,\upmu \text {M}$) and pH insensitive dye Sulfhorhodamine 101 (SR101, concentration: $15\,\upmu \text {M}$) were added to the crystallization solution.

The fluorescent dyes were excited at 488 nm and 552 nm. The fluorescent signal was detected with two Leica HyD detectors in photon counting mode. The fluorescent signals of R6G-EDA and SR101 were detected separately by setting the emission filters to 525 nm to 554 nm and 569 nm to 611 nm, respectively. The images were scanned sequentially line-by-line with a scanning speed of 100 Hz. Images were recorded with a resolution of 512 × 512 and a 3 × 3 pixel median filter was applied to all recorded images before further analysis.

The ratios of the fluorescent intensities of SR101 and R6G-EDA were used to analyze local changes in the pH. A standard curve was used to convert intensity ratios to pH values. Furthermore, a threshold was applied to all the local pH monitoring images, masking sand grains, calcite seeds and growing crystals gray in the local pH maps.

After the local pH monitoring the reaction was stopped with ethanol, and the sample cell was flushed with DI water.

### Raman microspectroscopy

Raman microspectroscopy was used to characterize the polymorph phase of the precipitated $\text {CaCO}_{3}$ crystals. For that the Ibidi sticky-Slide was removed from the polymer slide and the crystals on the polymer slide were analyzed using a Renishaw InVia Reflex Spectrometer. The measurement was performed with a 532 nm laser and a Leica 50X 0.75NA lens.

### Scanning electron microscopy

A Hitachi S-3400N scanning electron microscope (SEM) was used for characterizing the precipitated crystals. The polymer slides were sputter coated with a 10 nm layer of Pt/Pd (80/20) with a Cressington 208 HR sputter coater. The coated polymer slides were attached to the SEM stub with carbon tape.

## Results

### Sample cell: fabrication process

For studying time dependent processes, it is important that the seed crystals and sand grains are firmly attached to the substrate surface. The attachment process of calcite crystals is a rather straightforward process, as the crystals can be grown directly on the surfaces inside the sample cell. Attachment of sand on a substrate however is more challenging. Different methods were tested to form a stable arrangement of the sand grains inside the sample cell. The tested methods included: (1) Mounting sand grains between two microscope slides that were pressed together; (2) Using a thin layer of PDMS (Polydimethylsiloxan) on a slide to attach the sand during PDMS cross-linking; (3) Using a polymer slide that was heated above the glass transition temperature to firmly attach the sand to the slide surface. The best optical result and best attachment of the sand grains was achieved with the thermal attachment process that was further optimized to preserve optical quality of the polymer substrate. For that the sand was placed on the polymer slide and heated for 10 min at 200 °C (“Materials and methods”) inside an oven. To improve the attachment of the sand, a slight pressure was applied to the grains during the heating process. This can for example be achieved by placing a glass plate on top of the sand that is placed on the polymer slide.

### Precipitation in presence of calcite seeds

**Figure 2:**
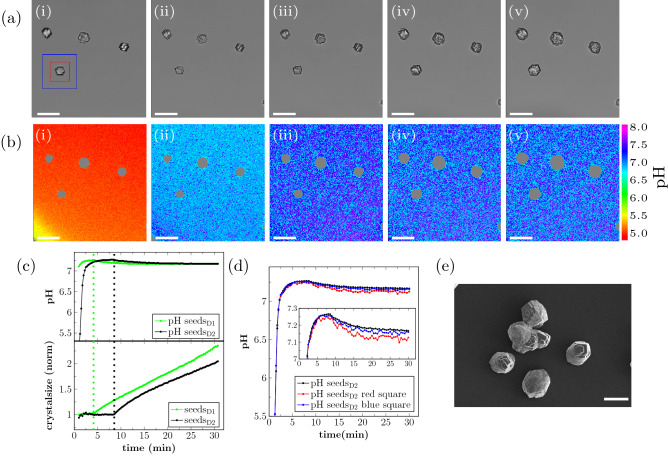
EICP reaction with calcite seeds present: (**a**) Brightfield images and (**b**) local pH monitoring after (i) 1 min, (ii) 2 min, (iii) 10 min, (iv) 20 min and (v) 30 min with sample seeds$_\text {D2}$. (**c**) Average pH and change of cross-sectional area of all the crystals for seeds$_\text {D1}$ and seeds$_\text {D2}$. (**d**) Local pH analysis of average pH for the red marked area in (**a**,i), the blue marked area in (**a**,i) and for the field of view of seeds$_\text {D2}$. (**e**) SEM micrograph of sample seeds$_\text {D2}$. The scale-bar in (**a**,**b**,**e**) is $50\, \upmu \text {m}$.

In the first part of the experiment the precipitation process in presence of calcite seeds was investigated. Two enzyme concentration were investigated and the starting concentrations of urea and calcium i the crystallization solution were 0.1 M and 122 mM, respectively (see “Materials and methods”). The precipitation process induced by the urease enzyme in the presence of calcite seeds is shown in Fig. 2. Brightfield images and local pH maps of the lower enzyme concentration are shown in Fig. 2a,b, respectively. In the initial phase of the reaction, a uniform, fast pH increase was observed in the sample (Fig. 2b, i-ii). This pH increase was caused by the urea hydrolysis reaction (see Eq. 1) and in the absence of DCS, pH would be expected to increase quickly above pH 8. In the presence of DCS, the crystallisation process stabilized the pH below 7.25. The calcite seeds on the polymer slides started to increase in size approximately 10 min after the reaction was started, caused by $\text {CaCO}_{3}$ precipitation. The pH value decreased by 0.2 units in close proximity to the growing seeds while the local pH in a location distant from the crystal seeds decreased by approximately by 0.1 units on the onset of precipitation. (Fig. 2b, iii-v). The local pH decrease in close proximity to the growing seeds can also be seen in Figure S1, Supplementary Information.

The pH decrease due to growth of the seeds could also be detected in the average pH of the field of view. The average pH of the images, calculated for each time-point, is shown in Fig. 2c. As shown here, a pH decrease was detected 8 min after the start of the reaction. That the pH decrease was caused by growth of the seeds could also be confirmed by comparing the average pH of the sample with the increase in cross-sectional area of all the crystals in the field of view during the experiment (Fig. 2c). The pH was measured for each pixel in the image. The average pH was determined by calculating the average of all pixels in one image. For sample seeds$_{\text {D2}}$ the average pH increased fast in the initial phase of the experiment until it reached a maximum value. During the phase of increasing pH, the cross-sectional seed size was approximately constant. The subsequent decrease in pH was found to coincide with the onset of crystal growth, detected as a steady increase in the size of the calcite seeds. This trend was observed for both of the investigated enzyme concentrations.

In Fig. 2c, the onset of crystal growth is marked with a vertical dashed line for both concentrations. As expected, the pH increase was faster and crystal growth started at an earlier time-point for the sample with the higher enzyme concentration (seeds$_{\text {D1}}$). The maximum average pH in the field of view of the experiment, as well as the rate of crystal growth measured as the slope of the increase in crystal size, was approximately the same for both samples. For both enzyme concentrations only growth of the calcite seeds was detected, and no nucleation of new calcite crystals on the polymer slide was observed.

The spatial distribution of pH was investigated further by comparing the average local pH in an area close to a calcite seed (Fig. 2a,i, red square) with a larger area around the same seed (Fig. 2a,i, blue square) and with the average pH in the field of view (Fig. 2d). During the initial phase of the experiment, the pH increase was the same for the red square, the blue square, and the field of view. The maximum measured pH in these areas was also the same. The subsequent pH decrease was found to be more pronounced for the smallest area around the seed (red square in Fig. 2a,i and red curve in d), showing that the pH decreased locally around the growing crystal. A corresponding pH evolution can be obtained for the other growing crystal in the field of view.

After a reaction time of 3 h the reaction was stopped by injecting ethanol, and the precipitated crystals were characterized with SEM (Fig. 2e). SEM micrographs showed that the precipitated $\text {CaCO}_{3}$ had a rhomohedral shape, which is typical for calcite. The calcite seeds could not be identified in the SEM micrographs indicating that the calcite seeds were overgrown with precipitated $\text {CaCO}_{3}$. The longest dimension of the crystals in the SEM micrograph was approximately ${50}\, \upmu \text {m}$.

### Precipitation in the presence of sand

Furthermore, the EICP reaction was investigated in the presence of sand grains. In the first part of the experiments in the presence of sand, a single injection of the crystallization solution in the sample cell was investigated by monitoring the precipitation process and local pH changes. Subsequent, the effect of multiple injections of the crystallization solution on the consolidation process was investigated.

#### Single injection

**Figure 3:**
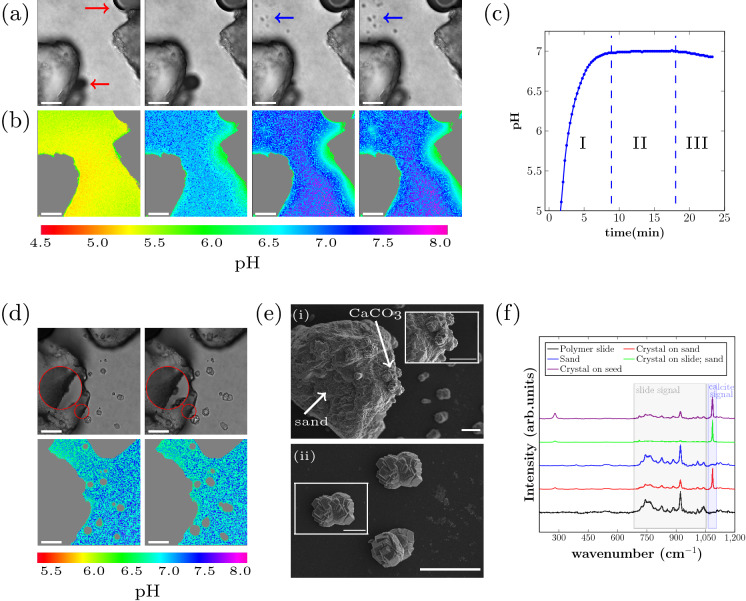
EICP reaction with sand present: (**a**) Brightfield images and (**b**) local pH monitoring after 2 min, 5 min, 20 min, 22 min for sample sand$_\text {D2}$. The images show the edges of two sand grains on the left and right side of the image, respectively. The sand grains and air bubbles are marked gray in the local pH maps. The air bubbles are marked with a red arrow in one optical micrograph. The precipitated crystals in (**a**) are marked with blue arrows. (**c**) Average pH of the local pH monitoring images shown in (**b**). (**d**) Optical micrographs (top) and local pH monitoring (bottom) of crystal growth on sand grain and on polymer slide after 30 min (left) and after 55 min(right) of the reaction. The magnified area in the optical micrographs shows a $\text {CaCO}_{3}$ crystal growing on the surface of the sand grain (note that a different position of the sample than in (**a**,**b**) is shown). (**e**) SEM micrographs of sample sand$_\text {D2}$. Sand grain with calcite crystals on the surface (top) and calcite crystals grown on the polymer slide (bottom). (**f**) Raman microspectroscopy of sample sand$_\text {D2}$ and seeds$_\text {D2}$. The scale-bars in (**a**,**b**,**c**,**e**) are $50\, \upmu \text {m}$ and $20\, \upmu \text {m}$ in the insert image of (**e**,ii).

Real time and in situ monitoring of the initial phase of the EICP reaction for sand$_\text {D2}$ is shown in Fig. 3a. Including sand grains in the sample cell results in a cell with more heterogeneity in the vertical dimension, since the sand grains are larger in size ($210\, \upmu \text {m}$ to $297\, \upmu \text {m}$) than the calcite seeds. Confocal laser scanning microscopy gives a 2D measurement in one chosen focal plane, so that only processes within or close to that focal plane can be detected. While for the seeded samples all precipitation processes take place within one focal plane, in the sand system, precipitation processes also take place outside the focal plane. At the start of the experiment it is not possible to know at which location and focal plane in the sample crystals will precipitate on the sand surface. Therefore, we chose to observe two different focal planes in these experiments: one approximately $25\, \upmu \text {m}$ above the polymer slide to monitor the initial phase of the reaction until the first crystals nucleated, and one close to the polymer slide on the bottom of the channel after crystals had nucleated.

To observe the initial phase of the EICP reaction, we chose the focal plane $25\, \upmu \text {m}$ above the polymer slide. In the optical micrographs (Fig. 3a) we observed $\text {CaCO}_{3}$ crystal growth on the polymer slide (dark spots at the top left corner after 20 min in Fig. 3a, not in focus). The nucleation on the polymer slide was not in the focal plane of the measurement, but the pH decrease due to nucleation on the slide surface could still be detected $25\, \upmu \text {m}$ above the slide. We observed that the pH in the field of view between the sand grains increased uniformly in the starting phase of the reaction (Fig. 3b), as expected from the urea hydrolysis reaction. Nucleation and growth of $\text {CaCO}_{3}$ on the polymer slide caused the pH to decrease locally, while the pH still increased between the sand grains.

In these experiments, we could also identify three stages in the pH evolution. The pH increased fast during the first 9 min of the reaction, due to hydrolysis of urea (Stage I). Subsequently, a phase of stable average pH was detected (Stage II). The pH started to decrease approximately 18 min after the start of the reaction (Stage III). This pH decrease coincided with the observed nucleation and growth of $\text {CaCO}_{3}$ on the polymer slide.

Another interesting observation was an air bubble (top right corner in Fig. 3a) that was trapped on one sand grain. We could detect that this air bubble resulted in a local pH decrease around the bubble. The locally lower pH can influence $\text {CaCO}_{3}$ precipitation and can therefore have implications for the homogeneity of the consolidated material.

30 min after the start of the reaction, we shifted the focal plane to a position just above the polymer slide surface. Here, we could monitor crystal growth on sand surfaces and on the polymer slide simultaneously. Crystal growth after 30 min and 55 min as well as the corresponding pH maps are shown in Fig. 3d, also showing a small air bubble was trapped on one sand grain. The smaller size of the $\text {CaCO}_{3}$ crystals on the sand surfaces indicate later nucleation than the crystals on the slide surface. A local pH decrease was detected in close proximity of the crystals growing on the polymer slide and on the edge of the sand grain (magnified area in the optical micrographs in Fig. 3d). The local pH differences caused by $\text {CaCO}_{3}$ crystals growing on sand surfaces that were not perpendicular to the line of view could not be detected with this method.

After a reaction time of 2 h the reaction was stopped by injecting ethanol, the sample cell was disassembled and the polymer slide was washed with DI water and dried for Raman microspectroscopy and SEM analysis. SEM micrographs of the sample (sand$_\text {D2}$) are shown in Fig. 3e. In Fig. 3e(i), $\text {CaCO}_{3}$ crystals on the surface of a sand grain are indicated by a white arrow. Crystals grown on the polymer slide are also visible. The insert shows a higher magnification of a $\text {CaCO}_{3}$ crystal grown at the sand surface. In Fig. 3e(ii) the $\text {CaCO}_{3}$ crystals grown at the polymer slide are shown with a higher magnification. Both the crystals grown on the sand surface and the crystals grown on the polymer slide have the same rhombohedral morphology, which is characteristic of calcite crystals.

Raman microspectroscopy (Fig. 3f) was additionally used to investigate the precipitated crystals from the different experiments. All the precipitated $\text {CaCO}_{3}$ crystals showed a Raman peak at 1085 cm$^{-1}$, which is characteristic for calcite. This indicates that the major part of the precipitated $\text {CaCO}_{3}$ crystals are calcite. Moreover, the polymer slide contributed to the Raman signal, but no characteristic peak for sand was detected with Raman analysis.

#### Multiple injections

The effect of multiple injections on the consolidation state of the sand was investigated by injecting the crystallization solution (sand$_\text {D1}$) one, four, and six times in the sample cell with sand grains present. The reaction time between each injection was 45 min and between each injection the channel was flushed with DI water (see “Materials and methods”). The precipitated crystals were characterized with SEM.

**Figure 4:**
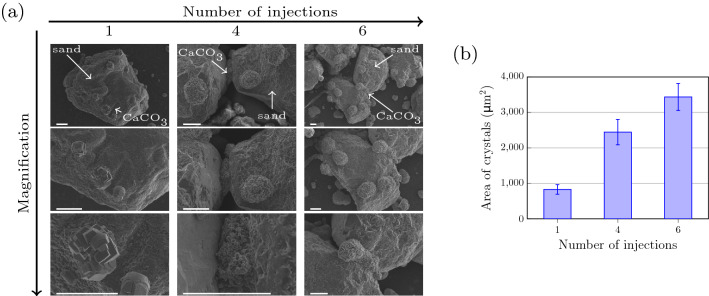
Multiple injection experiment with a urease concentration of ${13}\, \upmu \text {g}\,\text{L}^{-1}$ in the crystallization solution (sample sand$_\text {D1}$) in a sample cell with sand grains present. (**a**) SEM micrographs of sand sample cell after 1, 4, and 6 injections of the crystallization solution with different magnifications. The scale-bar is ${50}\, \upmu \text {m}$ in all SEM micrographs. Sand and $\text {CaCO}_{3}$ are marked with white arrows. (**b**) The cross-sectional area of $\text {CaCO}_{3}$ crystals and aggregates on the surface of the sand grains was measured from the SEM micrographs and is shown as a function of the number of injections.

SEM micrographs of calcite crystals on the surfaces of sand grains are shown after one, four, and six injections in Fig. 4a, with three different magnifications. In the single injection experiment we showed that during the reaction calcite crystals will precipitate at the surface of the sand grain and at the polymer slide surface. However, for biocementation only crystals on the sand surface are of interest. The precipitated crystals after one injection have a rhombohedral morphology, which is typical for calcite.

For further injections of the crystallization solution not only the sand grains, but also the calcite crystals need to be considered for the precipitation process. As seen in Fig. 2 the presence of calcite crystals is expected to influence the precipitation process and $\text {CaCO}_{3}$ precipitation is expected to result in the increase of size of the existing $\text {CaCO}_{3}$ crystals. For further injections we expect, for the chosen enzyme concentration, the growth of existing crystals, as observed in Fig. 2. After four injections larger aggregates of calcite were observed on the sand surfaces and between the sand grains. Calcite between the sand grains formed a connection between the grains. The aggregates had a spherical morphology with a rough surface. This trend continued also for a higher number of injections. After six injections the aggregates increased further in size. Also here, the calcite could fill the gap between the grains and connected them together.

The contact area of the calcite crystals and aggregates to the sand surface was analyzed by detecting the edges of the crystals in the SEM micrographs and measuring their size. The contact area was plotted as a function of number of injections in Fig. 4b, where it can be seen that the contact area of the crystals to the sand increases with increasing number of injections.

## Discussion

The presented sample cell is simple and fast to fabricate and allows to change experimental parameters such as sand type, grain size, and amount of sand as well as amount of calcite seeds and seed size. To show the principle and demonstrate the various characterization possibilities, which the presented sample cell offers we used EICP under stagnant conditions (no flow). However, the sample cell additionally has the option to cultivate bacteria and is therefore also suitable for MICP experiments and experiments can also be performed under non-stagnant conditions (flow). We have used a similar sample cell previously to monitor local pH changes during MICP precipitation without additional crystallization surfaces present and for the dissolution of $\text {CaCO}_{3}$ crystals with lactic acid^22^.

In the seeded experiments (Fig. 2) we observed that growth of the seeds resulted in a local pH decrease in close proximity to the seeds. Such local differences in pH cannot be detected in bulk measurements. The pH decrease caused by the onset of seed growth in MICP experiments has been reported previously, where we used a method measuring the average pH in small volumes in real time during the precipitation reaction^17^. With the presented sample cell in combination with confocal laser scanning microscopy we could now detect the pH decrease and simultaneously observe the growth of the seeds in real time and in situ.

Comparing precipitation with seeds (Fig. 2) and sand grains (Fig. 3) present showed: (1) The pH increase in the initial phase of the experiment was faster for samples with calcite seeds present and the maximum pH value was reached at an earlier time-point in the experiment. This was most likely caused by the fact, that the crystallization solution which was added to the sample cell was under-saturated in the beginning of the experiment, which resulted in a partial dissolution of the calcite seeds^17^. Due to the injection of a low pH solution that is under-saturated with respect to $\text {CO}_{3}^{2-}$, a partially dissolution of the calcite seeds was expected.

(2) Calcite growth started at an earlier time-point in the seeded experiments compared to calcite nucleation in experiments with sand present (Figs. 2c and 3c). No nucleation on the polymer slide was detected for seeded samples. For the sand sample cell a stable pH region was observed before calcite nucleation, while for seeded experiments seed growth started fast after the maximum pH was reached in the sample. The presence of foreign surfaces in heterogeneous nucleation can result in the reduction of the energy barrier necessary for nucleation. This reduction of the energy barrier depends on the surface tensions between the crystal phase, the liquid phase and the foreign solid substance. Since the calcite seeds have the same surface chemistry as the crystal phase no energy barrier for nucleation exists^12^. Therefore, the seed growth started right after a sufficient supersaturation was established. For seeded samples (Fig. 2c), onset of the crystal growth as determined from the crystal size visible in the optical microscope, coincides with the time point at which the maximum pH is reached. It is likely that the grow starts before that point, however the growth rate is too low to be detected. In the experiments with sand present we found that the pH stabilized first before calcite nucleated. Calcite nucleated first on the polymer slide, and only later on sand surfaces. The pH was locally lower when calcite nucleated on the polymer slide than when it nucleated on the sand surface. Together with ongoing modelling work that involves geochemical approaches, changes in the pH combined with enzyme activity should be sufficient to fully describe the chemical processes in the experimental system under investigation.

The enzyme activity was not directly determined before each experiment, however the enzyme stock solution was prepared in a reproducible manner (see “Materials and methods” section) to ensure comparability of the experiments.

The air bubbles trapped on the sand grains caused a locally decreased pH, due to $\text {CO}_{2}$ and $\text {NH}_{3}$ exchange with the surrounding solution. The air bubble itself is masked in the pH maps (Fig. 3b,d), however the locally decreased pH can be observed along the sand grain surface.

The suitability of sand as nucleation sites in heterogeneous nucleation has been investigated previously. Lioliou et al.^23^ compared the nucleation of $\text {CaCO}_{3}$ in the presence of calcite and quartz seeds, and found that quartz seeds compared to calcite seeds could hardly act as nucleation surface for heterogeneous nucleation. In our experiments we could show that the sand grains, in absence of calcite crystals, acted as nucleation sites and we observe calcite nucleation and growth on the sand surface in real time and in situ (Fig. 3e).

It was suggested previously that calcite is the most suitable polymorph for a stable and permanent biocementation^24^. For MICP experiments it has also been reported that the calcium source can influence the polymorph selection^25^. With the presented sample cell we could observe that with DCS as a calcium source in EICP experiments mainly calcite crystals were formed.

The size and the distribution of the calcite crystals within the granular medium are important parameters for an effective biocementation process. In order to form stable connections between the sand grains, it is important that a significant amount of the calcite precipitates close to particle–particle contacts^26^. The calcite aggregates also should be large enough to form a good connection with the sand surface. In the multiple injection experiment (Fig. 3), we found that the contact area between aggregates and sand grains increased with an increasing number of injections (Fig. 4b), promoting effective connections between the sand grains (Fig. 3a).

## Conclusion

In this paper, we have introduced a microscope sample cell which allows the study of $\text {CaCO}_{3}$ precipitation processes for the application of biocementation in real time and in situ in the presence of calcite seeds and sand grains. EICP by urea hydrolysis has been used as a showcase for the various experimental methods and characterization possibilities that this sample cell offers. Because precipitated crystals can be characterized with Raman microspectroscopy as well as SEM without further processing of the sample, this setup makes it possible to study the consolidation process of sand thorough. The maps of local pH during the precipitation process can be valuable for benchmarking numerical biogeochemical pore-scale models.

## Supplementary Information

Supplementary Information.

## Data Availability

All the data used in this study are available from the corresponding author upon request.
