# Supplementary material for: A sample cell for the study of enzyme-induced carbonate precipitation at the grain-scale and its implications for biocementation
Source: Sci Rep. 2021 Jul 1;11:13675. doi: 10.1038/s41598-021-92235-7 (PMC8249643; doi:10.1038/s41598-021-92235-7)
Supplement: Supplementary file 1 — Supplementary Information. [file 41598_2021_92235_MOESM1_ESM.pdf]

Supplementary Information: A sample cell for the study of enzyme-induced carbonate precipitation at the grain-scale and its implications for biocementation

Jennifer Zehner[1,*], Anja Røyne[2], and Pawel Sikorski[1]

[1]Department of Physics, Norwegian University of Science and Technology, Trondheim, Norway
[2]The Njord Centre, Department of Physics, University of Oslo, Oslo, Norway
[*]jennifer.s.zehner@ntnu.no

ABSTRACT

Biocementation is commonly based on microbial-induced carbonate precipitation (MICP) or enzyme-induced carbonate precipitation (EICP), where biomineralization of $CaCO_3$ in a granular medium is used to produce a sustainable, solid, and concrete-like material. The successful implementation of biocementation in large-scale applications requires detailed knowledge about the micro-scale processes of $CaCO_3$ precipitation and grain consolidation. For this purpose, we present a microscopy sample cell that enables real time and *in situ* observations of the precipitation of $CaCO_3$ in the presence of sand grains and calcite seeds. In this study, the sample cell is used in combination with confocal laser scanning microscopy (CLSM) which allows the monitoring *in situ* of local pH during the reaction. The sample cell can be disassembled at the end of the experiment, so that the precipitated crystals can be characterized with Raman microspectroscopy and scanning electron microscopy (SEM) without disturbing the sample. The combination of the real time and *in situ* monitoring of the precipitation process with the possibility to characterize the precipitated crystals without further sample processing, offers a powerful tool for knowledge-based improvements of biocementation.

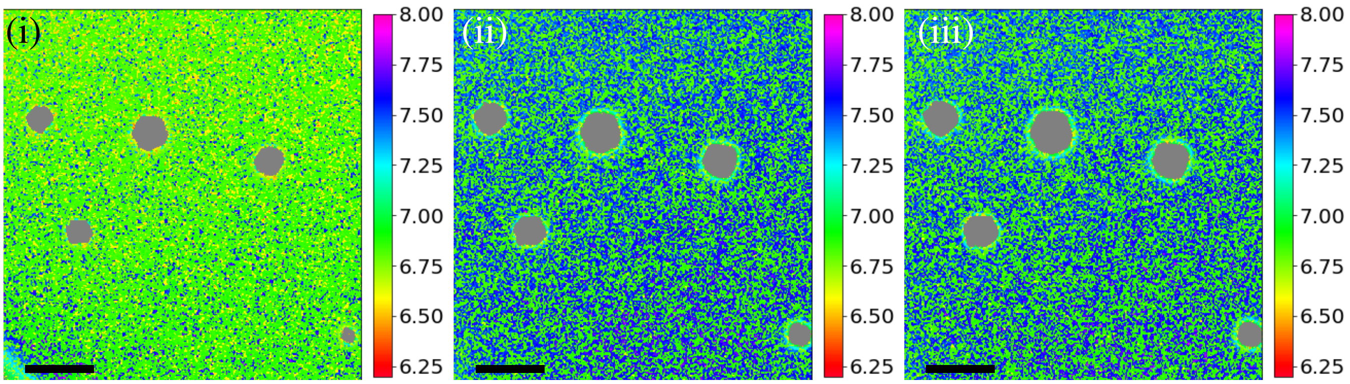

Figure S1. EICP reaction with calcite seeds present: local pH monitoring after (i) 2 min, (ii) 10 min, and (iii) 20 min for seeds$_{D2}$. The scale-bar in (a,b,e) is 50 µm.
